# Integration of Precision Medicine into ERAS Pathways: A Conceptual Framework, Current Feasibility and Challenges

**DOI:** 10.3390/jpm16070366

**Published:** 2026-07-04

**Authors:** Berkan Aliev, Boyko Atanasov

**Affiliations:** 1 Department of Anesthesiology, Emergency and Intensive Care Medicine, Medical University of Plovdiv, 4002 Plovdiv, Bulgaria; 2 Clinic of Anesthesiology and Intensive Care, University Hospital St. George, 4000 Plovdiv, Bulgaria; 3 Department of Propedeutics of Surgical Diseases, Section General Surgery, Medical University of Plovdiv, 4002 Plovdiv, Bulgaria; 4 Department of General Surgery, Multiprofile Hospital for Active Treatment “UniHospital”, 4500 Panagyurishte, Bulgaria

**Keywords:** Enhanced Recovery After Surgery (ERAS), precision medicine, precision perioperative care, artificial intelligence, predictive analytics, digital health, multi-omics, risk stratification

## Abstract

Enhanced Recovery After Surgery (ERAS) pathways have improved perioperative outcomes by standardizing evidence-based interventions across the surgical continuum. However, substantial variability in postoperative recovery persists, even within well-implemented ERAS programs. This heterogeneity reflects differences in clinical risk, functional reserve, biological response to surgical stress, treatment responsiveness, and contextual factors that are not fully captured by uniform protocols. Precision medicine provides a potential framework for refining ERAS by integrating patient-specific data into perioperative risk assessment, intervention selection, patient monitoring, and recovery planning. Nevertheless, most precision medicine tools remain insufficiently validated for routine ERAS implementation, and their clinical utility is limited by heterogeneous evidence, data integration challenges, costs, workflow complexity, and equity concerns. Future progress will require prospective validation, pragmatic implementation studies, interoperable data systems, and evaluation of patient-centered outcomes. This narrative review examines the emerging role of precision medicine tools in perioperative practice and proposes an idealized conceptual model of “precision ERAS” in which standardized evidence-based care is preserved as the foundation, while selected interventions are adapted according to individual risk, biological phenotype, and recovery trajectory.

## 1. Introduction

Surgery remains a cornerstone of treatment for a wide range of diseases, with more than 300 million procedures performed worldwide each year [1]. Despite advances in surgical and anesthetic techniques, perioperative morbidity and mortality remain substantial, and surgery-related events continue to represent a major global health burden [2]. Importantly, surgery constitutes a controlled physiological insult, and postoperative outcomes are determined not only by technical factors but also by the magnitude and regulation of each individual patient’s stress response to surgical trauma [3].

Enhanced Recovery After Surgery (ERAS) programs were developed to attenuate patients’ stress response through standardized, evidence-based perioperative care. Since their introduction, ERAS pathways have consistently demonstrated reductions in postoperative complications, hospital length of stay, and healthcare costs across multiple surgical disciplines [4]. However, despite these population-level benefits, substantial inter-patient and inter-center variability in postoperative recovery persists. Even within well-implemented ERAS programs, patients undergoing similar procedures may experience markedly different recovery trajectories and complication risks [5,6]. This persistent heterogeneity suggests that uniform perioperative pathways, while effective on average, may not adequately account for biological and physiological differences between individual patients.

The surgical stress response includes integrated neuroendocrine, inflammatory, immune, and metabolic changes and can be modulated by anesthetic, analgesic, and perioperative interventions [7]. Emerging perioperative literature further suggests that the magnitude and trajectory of this response may vary between patients, providing a rationale for studying patient-specific biological and physiological profiles within ERAS [8]. While ERAS pathways are designed to optimize recovery through standardized protocols, they are built on the premise that most patients will benefit from similar approaches. However, new discoveries in systems biology and perioperative physiology highlight opportunities to further enhance these pathways by incorporating more individualized patient care [9].

Precision medicine may provide a framework to address this limitation by tailoring prevention, monitoring, and treatment strategies to individual patient characteristics. Precision medicine emphasizes data-driven personalization rather than one-size-fits-all protocols and is already being integrated into or showing considerable promise for fields such as oncology, cardiology, and critical care [9]. Application to perioperative care is particularly attractive as surgery represents a predictable, time-limited physiological and somatic insult within a data-rich and biologically defined setting, across highly heterogeneous patient cohorts. Within ERAS protocols, such an approach could theoretically support more appropriate matching of selected interventions to patient-specific risk, timing, and recovery needs.

This narrative review examines the current integration of individualized care within ERAS systems and explores contemporary and future opportunities to transform static, population-based protocols into dynamic, truly personalized perioperative care systems.

## 2. Materials and Methods

The narrative review approach used in this study was deemed most suitable as it allows for synthesis of heterogeneous evidence spanning perioperative physiology, clinical risk stratification, biomarker research, digital health technologies, and predictive analytics.

Nonetheless, a focused literature search was performed across major biomedical databases (PubMed/MEDLINE and Embase). The literature search focused on articles published in English from January 2000 to February 2026 and used a combination of keywords and MeSH terms, including “Enhanced Recovery After Surgery” or “ERAS”; “precision medicine” or “personalized medicine”; “perioperative care” or “perioperative medicine”; “risk stratification,” “frailty,” “biomarkers,” and “pharmacogenomics”; and “goal-directed therapy,” “digital health,” “wearables,” and “predictive analytics”. The reference lists of key review articles and consensus statements were also screened to identify additional relevant publications not captured by the initial database searches.

Artificial intelligence (AI)-assisted tools (LeapSpace™ (web-based research-grade AI workspace; Elsevier B.V., Amsterdam, Netherlands; accessed 4 March 2026,ChatGPT (GPT-5.5 Thinking; OpenAI OpCo, LLC, San Francisco, CA, USA), and Microsoft 365 Copilot (Microsoft Corporation, Redmond, WA, USA; Microsoft 365 Apps, accessed 1 May 2026) played a supportive role in conducting this narrative review, enhancing the efficiency and consistency of the literature synthesis. AI-assisted tools were used for literature exploration, literature organization, drafting, language refinement, and manuscript editing. All AI-generated outputs were critically reviewed and verified by the authors, who take full responsibility for the accuracy, integrity, and originality of the manuscript.

A formal inclusion and exclusion process was not applied; instead, articles were selected based on their relevance to the following domains:

1. Established ERAS principles and contemporary adaptations;
2. Risk stratification strategies in perioperative care;
3. Precision medicine tools with potential relevance to perioperative care (biomarkers, genomics, and digital health);
4. Evidence supporting individualized and adaptive perioperative management.

Emerging or exploratory studies were selectively included to illustrate developing concepts with explicit acknowledgment of limitations.

Because this was not a systematic or scoping review, the search strategy was intended to support conceptual synthesis rather than exhaustive evidence retrieval. Therefore, selection bias is an inherent methodological limitation; this review is transparent regarding its scope and interpretive nature, and it does not claim to achieve systematic completeness. Its purpose is not to establish clinical practice recommendations, but to synthesize relevant concepts and propose a framework to guide future research on the integration of precision medicine into ERAS pathways.

## 3. From Standard ERAS to Precision ERAS

**Figure 1:**
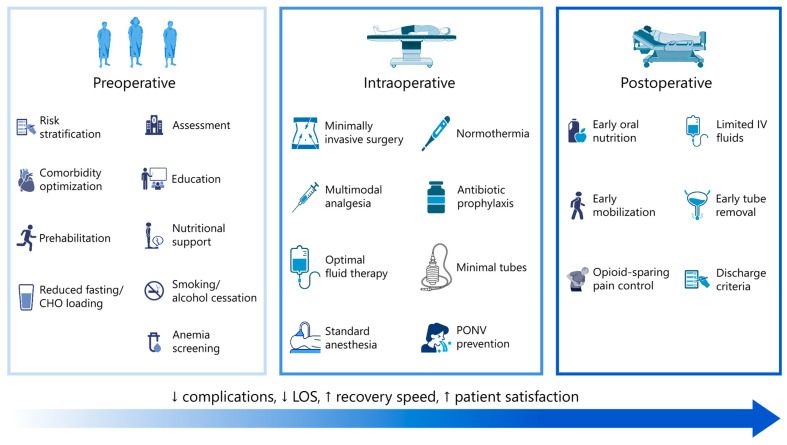
Framework of a standard ERAS pathway, with illustration of key perioperative components. Created in https://BioRender.com, acessed 17 June 2026.

Historically, the ERAS methodology was based on the principle of actively managing and mitigating surgical stress to optimize patient outcomes. In the 1990s, Henrik Kehlet hypothesized that using a structured approach in perioperative care would attenuate the risks associated with surgery [10]. In addition, Bardram and associates observed that combining minimally invasive surgery, multimodal pain management, early oral nutrition, and early mobilization allowed patients to be safely discharged 1–2 days earlier than with the care they were previously receiving [11]. This led to the design of the ERAS framework, which covers the period from the first idea of surgery to the last follow-up. Every ERAS care pathway is constructed with 15–25 different perioperative care elements, which have been independently shown to be beneficial and to improve surgical outcomes. The interventions are conceptually categorized through the stages of the perioperative period: preoperative, intraoperative, and postoperative. ERAS care pathways are constructed by combining general ERAS and surgery-specific elements. General ERAS care interventions include patient education, comorbidity optimization, anemia screening and treatment, shorter length of fasting, carbohydrate loading, minimally invasive surgery, standardized anesthetic management, multimodal opioid-sparing analgesia, optimal fluid management, postoperative nausea and vomiting (PONV) prophylaxis, early mobilization, early enteral nutrition and early removal of drainages, urinary catheters, and naso-gastric tubes (Figure 1) [12].

However, since the creation of the concept and its more widespread adoption, questions of its sufficiency and adequacy have risen [13,14]. Although RCTs have consistently proven that ERAS care protocols can shorten the hospital length of stay and lower the risk of overall complications, systematic reviews and meta-analyses have shown that it is hard to achieve a high level of compliance with all of the proposed elements, which leads to substantial inter-center variability. Furthermore, residual inter-patient individual heterogeneity is suggested to persist even in studies with above 70–80% compliance with ERAS principles [6]. In addition, distinct types of surgical procedures can carry markedly different surgical stress response magnitude and trajectory, based not only on the extent of surgical trauma but also on the interplay between it and the patient’s own biology [8]. These considerations have led to the adoption of a more stratified risk-adapted approach in perioperative patient care with the acknowledgment that older and, in particular, frail patients, as well as those with significant comorbidities need a more nuanced management approach. The result is a conceptual shift from the “one-size-fits-all” strategy to a more individualized strategy. While stratification by age, comorbidity, and dependence level has shown some benefit, biological variability is still not considered [15,16]. Consequently, patients within the same risk category differ in inflammatory response, metabolic reserve, functional resilience, and psychosocial characteristics. Henrik Kehlet argues that an inherent limitation of ERAS research is that “There is rarely a discussion about patient-specific factors (such as specific co-morbidities, high-pain responders, specific pharmaceutical agents needing attention in perioperative medicine)” [13,17]. This underscores the importance of continuing to improve perioperative care by addressing individual patient characteristics.

The principles and practice of precision medicine have led to a revolution in medical care by combining data from genetic signatures, inflammatory biomarkers, metabolic responses, and functional reserve, creating the means to address individual patient needs and to transition from population-level benefit to individual patient benefit [9]. At the same time, advances in biomedical science and digital health have helped to reveal that patients show biological heterogeneity in perioperative recovery as well [18,19,20]. Consequently, the persistence of postoperative complications, delayed functional recovery, and unplanned readmissions—even within well-implemented ERAS programs—can be partially explained by biological variability, which traditional clinical risk assessment tools and standard pathways are not designed to address. Thus, precision perioperative care may represent the logical continuation of improving surgical outcomes by extending population-level benefit toward individual-level optimization.

Considering that the ERAS model has been transformative and an essential part in evidence-based perioperative care, precision medicine tools may extend ERAS principles by tailoring perioperative care elements to the individual patient’s biological, functional, and contextual profile. The “precision ERAS” model would use ERAS as a foundational structure of evidence-based practices, while precision medicine would refine the selection, timing, intensity, and combination of care elements.

For clarity, the present review distinguishes three levels of adaptation within ERAS perioperative care. “Individualized ERAS” refers to pragmatic modification of standard ERAS care according to clinical judgment, contraindications, patient preferences, and perioperative events. “Risk-adapted ERAS” refers to systematic adjustment of pathway intensity according to recognized clinical risk strata, such as frailty, sarcopenia, anemia, nutritional risk, cardiopulmonary reserve, emergency surgery, or predicted morbidity. “Precision ERAS,” as used in this review, is a narrower and more investigational concept: ERAS adaptation guided by validated patient-specific biological, molecular, digital, or predictive data, with explicit links between the identified phenotype or risk signal and a defined perioperative action. This distinction is necessary because much of what is currently described as personalized, tailored, or individualized ERAS in the literature represents high-quality risk-adapted clinical care rather than precision medicine in the strict sense.

## 4. Current State of Clinical Individualization Within ERAS

The concept of ERAS has shown notable evolution from the development of the “fast-track” philosophy in colorectal and cardiac surgery to contemporary practice. Standardized, protocol-driven care bundles have introduced risk-adapted, physiology-targeted interventions into their routine implementation.

### 4.1. Risk Stratification

Risk assessment tools assessing frailty, nutritional status, anemia and comorbidity burden are recommended to tailor perioperative planning, management and decision-making [21]. While risk-adapted care represents a step toward a more individualized approach, the tools in use exhibit some inherent limitations. They remain largely population-based and static and as such they have limited utility in guiding intervention. Most models predict outcomes such as mortality, morbidity, and readmission, without identifying the mechanisms driving those risks [22]. An evidence review by the National Institute for Health and Care Excellence in 2020 reported that the most commonly used risk assessment tools—POSSUM, P-POSSUM, NSQIP, and ASA—show a fair level of accuracy for mortality prognosis (median c-statistic > 0.8) but notable inconsistency for predicting morbidity [23]. This discrepancy could be partly attributed to the primary focus of risk assessment tools on comorbidities and not on physiological reserve or biological and molecular characteristics, and to the limited procedure-specific granularity, i.e., lack of consideration of the surgical technique, operative duration, or intraoperative events. In an observational study by Rebecka Ah et al., P-POSSUM showed improved sensitivity and specificity when adjusted for ASA status, age, surgical indication, and procedure [24]. These findings suggest that more individualized risk assessment may improve predictive performance.

### 4.2. Perioperative Anemia

According to recent guidelines, routine anemia screening with targeted iron supplementation or erythropoiesis stimulation, based on laboratory values and urgency of surgical procedure, is strongly emphasized. This targeted correction of a modifiable risk could be considered an example of integrated biomarker-based management strategy [21].

### 4.3. Perioperative Fluid Management

Fluid and hemodynamic management within ERAS has evolved substantially toward more individualized perioperative care. Rather than supporting a uniform restrictive or liberal approach, contemporary ERAS guidance emphasizes avoidance of both fluid overload and hypovolemia, balanced maintenance of euvolemia, and selective use of hemodynamic monitoring or goal-directed fluid therapy (GDFT) in patients with high surgical or patient-related risk [21,25]. Although studies examining GDFT-based protocols within well-implemented ERAS pathways have shown limited benefit in outcome improvement for healthy low-risk patients, these protocols are considered valuable for managing patients with high combined individual and surgical risk. GDFT uses real-time dynamic physiologic data, thereby directly influencing clinical and therapeutic decision-making [26]. As treatment and timing are continuously adapted based on patient-specific physiological signals, GDFT could be viewed as one of the closest concepts to a precision approach within ERAS.

### 4.4. Analgesia

Multimodal opioid-sparing analgesia is an integral part of the ERAS philosophy, as it creates optimal conditions for early ambulation, early nutrition, stress modulation, and enhancement of gut recovery [10]. Published data show that analgesic strategies are commonly individualized and clinicians routinely adjust drug selection, dosing, and regional techniques according to factors such as age, renal function, and previous adverse reactions and type of surgical procedure [27,28]. This practice somewhat reflects an individualized approach grounded in patient variability rather than protocol uniformity, but it is still based on clinical observations and expertise rather than on sound biological or behavioral characteristics.

### 4.5. Prehabilitation, Mobilization, and Nutrition

Other core ERAS principles include preoperative prehabilitation, early postoperative mobilization, and perioperative nutrition [21]. Their implementation is commonly tailored to functional profile and milestones, nutritional requirements, tolerance of oral diet, and personal targets, with dynamic adjustments made based on clinical progress and recovery patterns rather than traditional timelines. Recently, in an RCT by Cambriel et al., a personalized prehabilitation program was able to modulate and dampen the inflammatory changes associated with surgery and postoperative complications when compared to a standard protocolized prehabilitation [29]. However, personalization in the study was driven primarily by clinical judgment rather than by comprehensive biological profiling, and it focused more on individualized support rather than dynamic interventional modification.

In summary, contemporary ERAS pathways already incorporate precision-aligned concepts such as risk stratification, targeted optimization of modifiable risk factors, dynamic physiology-based management, and individualized postoperative recovery targets and goals. However, clinical individualization based on population-derived risk assessment and personal judgment often falls short in truly capturing patient-specific risk factors and physiological variability necessary for optimal perioperative practice. This is where true biology-grounded precision medicine could potentially add benefit by linking individual biological signals to specific, actionable perioperative decisions.

## 5. Emerging Precision Medicine Tools Relevant to ERAS

ERAS has reshaped perioperative practice by creating the grounds for standardization and minimization in variability of care; nevertheless, embedding precision medicine tools into ERAS pathways could provide the means to dynamically assess and tailor the recovery process to individual patients’ needs. However, large volumes of data would need to be continuously analyzed to uncover the subtle interactions between genetic and metabolic signatures and determine how they are influenced by the external modification of surgery, anesthesia, and comorbid illness. This would make practical applicability of “precision ERAS” limited and cumbersome and would risk losing fidelity and compliance. With the introduction of the Big Data concept, machine learning, and other artificial intelligence (AI) modalities, perioperative care has moved closer to personalized care, as these tools create the basis for research and optimized workflow integration [30].

This section examines precision medicine tools which have shown some relevance and beneficial potential to perioperative practice. It also provides information on their current evidence support and how they could eventually be integrated into functioning ERAS pathways. Categorically, they are grouped into genomics and pharmacogenomics, proteomics and metabolomics, digital health and wearable technologies, and AI and predictive analytics.

### 5.1. Genomics and Pharmacogenomics

Genetic variation constitutes one of the foundational components of precision medicine. Genome-wide studies provide the molecular information necessary for targeted therapies, optimized drug choice, early and accurate disease detection, and enhanced risk prediction and support multi-omics approaches that further refine patient stratification. Genomics-enabled precision medicine is increasingly influencing modern healthcare [31,32]. In the context of perioperative care, genomic research has primarily shown promise in three interconnected domains: genomic risk assessment, pharmacogenomics, and the genomics of surgical stress response variability. However, the maturity of evidence and clinical actionability in these domains differ substantially and show limited current applicability.

The perioperative period provides a useful model for studying gene–environment interactions, because surgical trauma represents a relatively predictable physiological challenge. Evidence suggests that perioperative variability in postoperative recovery could be partly explained by complex interaction of static genome polymorphism, post-translational protein modification, and epigenetics. Although available data remains largely observational with limited practical translation into clinical practice, some specific genotypes have been reported to be associated with a variety of perioperative adverse events, such as myocardial infarction and atrial fibrillation, neurocognitive dysfunction, renal injury, postoperative thrombosis, perioperative lung injury, and sepsis [33]. For instance, genetic markers such as APOE ε4, TREM2, TOMM40, and METTL3 have been implicated in the pathogenesis of perioperative neurocognitive disorders [34]. In addition, single-nucleotide polymorphisms on chromosomes 9 and 14 have shown an association with sepsis and infectious complications [35,36]. In this context, genomic risk profiling offers a biologically plausible approach to identifying patients with increased vulnerability to postoperative complications, but its perioperative utility remains insufficiently tested in randomized studies.

Pharmacogenomics studies inheritable genetic variants that contribute to variations in drug efficacy and safety regarding both pharmacokinetic and pharmacodynamic interactions [33] and is a well-recognized tool in the arsenal of precision care. However, its current application in perioperative care is limited to explaining adverse events—such as malignant hyperthermia (RYR1/CACNA1S) and prolonged apnea after muscle relaxation (butyrylcholinesterase deficiency) [33]. Nevertheless, variations in anesthetic and analgesic requirements, antithrombotic and vasoactive medications have also shown relevance to perioperative practice [37]. The most widely studied pharmacogenetic marker, particularly applicable to perioperative care, is the microsomal enzyme family of cytochrome P450D6 (CYP2D6), which is implicated in phase I drug metabolism of many therapeutic substances and is of particular importance to codeine, tramadol, and ondansetron. Genomic studies have uncovered four types of CYP2D6 activity phenotypes: ultrarapid metabolizers, extensive metabolizers, intermediate metabolizers, and poor metabolizers [33]. A recent hybrid pragmatic type 2 study showed that CYP2D6-guided postoperative management was feasible and reduced postoperative opioid use without compromising analgesia [38]. Senagore et al. found that pharmacogenomic-guided analgesia within an ERAS pathway lowered pain scores, lowered opioid consumption by 50%, and reduced the incidence of analgesic-related side effects [39]. However, pilot findings from the first phase of the prospective ImPress trial have shown that although providers accessed pharmacogenomic data for 58% of surgical patients, genomically guided prescribing remained infrequent, which suggests feasibility and workflow barriers [40]. Integration of pharmacogenomic signatures into ERAS pathways could theoretically enable precise selection and dosing of perioperative medications and analgesic techniques to improve efficacy and reduce adverse events; however, the practical evidence base, mostly regarding feasibility, is still in its early stages. Although pharmacogenomics shows clinical promise, current adoption in perioperative care is limited by the need for broader multicenter validation, assessment of cost-effectiveness, and demonstration of improvement in postoperative outcomes beyond standard procedure-specific multimodal analgesia.

Genetic influences on the surgical stress response’s magnitude and trajectory are also recognized, although their perioperative clinical applicability remains largely unclear. Environmental factors, such as the extent of surgical injury and perioperative care, can interact with polygenic architecture and dynamic molecular remodeling to influence the regenerative process [33,41]. Several biologically plausible pathways have therefore been proposed as candidates for perioperative research, including HPA-axis regulation [33], immune-inflammatory signaling [41,42], coagulation [43], and pain-related pharmacogenomic pathways [44]. For example, inflammatory gene polymorphisms have been associated with postoperative morbidity after lung resection [42], whereas catechol-O-methyltransferase polymorphisms have been linked to postoperative opioid consumption [44]. Analyses of experimental and observational data have also revealed epigenetic changes relevant to perioperative biology. Major surgery has been associated with acute DNA methylation changes in immune-response pathways [41], while epigenetic mechanisms have been discussed in relation to chronic postsurgical pain [45], and postoperative delirium-related non-coding RNA profiles [46]. These findings support the possibility of modifiable perioperative biology, but they remain insufficient to guide routine precision ERAS interventions without further validation.

However, the current level of evidence supporting the use of genomics in perioperative care seems to have a strong proof of concept but rather broad and uncertain implementation power. The most mature domain is pharmacogenomics, where a clear mechanistic link and reproducible associations have been demonstrated, particularly for CYP2D6-guided opioid prescribing. In addition, further support has been documented by the CPIC guidelines, which provide therapeutic recommendations for CYP2D6, OPRM1, and COMT genotypes and selected opioid therapy, including codeine and tramadol [47]. In contrast, evidence from the studies included in this review shows that the domains of risk assessment and recovery trajectories rely mostly on observational, heterogeneous, and underpowered studies. We could not retrieve large multicenter randomized trials showing that genetic-guided perioperative interventions impact clinical or patient-centered outcomes. Consequently, integration of genomic data into individualized perioperative care is currently limited by a modest effect size, complexity, and clinical interpretability.

### 5.2. Proteomics and Metabolomics

Proteomics and metabolomics provide a dynamic view of the perioperative biological state and may complement more static genomic information. While genomic data describe biological predisposition, proteomic and metabolomic profiles may better reflect the current state of inflammation, immune activation, endothelial injury, mitochondrial function, tissue damage, metabolic stress, and early organ dysfunction [48,49]. In the context of ERAS, this is conceptually attractive because postoperative recovery is determined not only by preoperative risk, but also by the evolving biological response to surgical trauma, anesthesia, fluid therapy, analgesia, nutrition, and mobilization [20,49].

However, the clinical maturity of these tools varies considerably. At present, most perioperative proteomic and metabolomic studies remain observational, exploratory, and primarily prognostic rather than interventional. A scoping review of metabolomic profiling in surgery identified 47 studies, most of which were retrospective, small, and heterogeneous in sample type, analytical platform, and outcome definition; the authors concluded that metabolomics has diagnostic and prognostic potential but requires methodological standardization before routine clinical translation [50]. Therefore, these techniques should currently be framed as promising tools for biological phenotyping and hypothesis generation.

The most clinically advanced example of a proteomic or biomarker-guided perioperative strategy involves postoperative acute kidney injury prediction using urinary cell cycle arrest markers, particularly tissue inhibitor of metalloproteinases-2 and insulin-like growth factor-binding protein 7, expressed as [TIMP-2]·[IGFBP7]. These markers do not represent broad proteomics, but they provide a useful proof of principle for precision perioperative care because they link a measurable biological signal to a defined preventive intervention. In the BigpAK study, early biomarker-based identification of patients at high risk of acute kidney injury (AKI), followed by implementation of a KDIGO kidney protection bundle, reduced AKI severity and postoperative creatinine increase after major surgery [51]. More recently, BigpAK-2 evaluated a biomarker-guided preventive strategy to prevent AKI after major surgery and reported a reduction in moderate or severe AKI within 72 h of surgery [52].

Other studies also support the value of perioperative biomarkers for earlier detection of organ stress, but their actionability is more limited. For example, the MAYDAY trial used [TIMP-2]·[IGFBP7] to assess renal tubular stress in patients undergoing autologous breast reconstruction who were randomized to liberal or restrictive fluid therapy. Restrictive fluid management was associated with higher immediate postoperative renal stress biomarker levels. However, the trial was small, monocentric, and not designed to test whether the biomarker-triggered intervention improves clinical outcomes [53].

Proteomic profiling has also been studied in relation to postoperative inflammation and delirium. In a prospective cohort of 118 older elective surgical patients, perioperative multiplex profiling of vascular and inflammatory proteins showed that surgery-induced inflammatory changes, including CCL2 release, were associated with postoperative delirium, particularly after cardiac surgery [54].

Metabolomics has similarly shown promise for predicting recovery trajectories. In colorectal cancer surgery, blood metabolomic profiling in 48 patients undergoing goal-directed fluid therapy identified metabolites associated with delayed versus enhanced gastrointestinal recovery, and a support vector machine model was proposed to predict postoperative gastrointestinal function [55]. Nevertheless, the authors of this narrative review were unable to identify any studies that provide external validation or show the clinical utility of interventions guided by metabolomic profiles in improving recovery, reducing postoperative ileus, or shortening the hospital length of stay.

The included studies suggest that proteomic and metabolomic approaches may help characterize biological vulnerability earlier than the use of conventional clinical markers and support more precise postoperative surveillance, renal protection, nutritional strategies, inflammatory modulation, and escalation or de-escalation of care. However, their future integration into perioperative care will require prospective multicenter validation, standardized sampling and analytical methods, predefined decision thresholds, demonstration of clinical utility and cost-effectiveness, and evaluation of whether biomarker-guided interventions preserve ERAS fidelity and improve patient-centered outcomes.

### 5.3. Digital Health, Wearable Technologies, and Telemedicine

Recent advances in digital health platforms, wearable technologies, and telemedicine have created new opportunities for continuous perioperative monitoring, patient engagement, and early prevention of complications. Recent systematic evidence has outlined five areas of potential benefits in integrating mobile applications into perioperative care: patient education and guidance; improved communication; pain monitoring and control; monitoring of complications; and enhancing patient support, satisfaction, and safety [56]. Additionally, D’Ambrosio et al. suggest that ERAS mobile applications may improve adherence to perioperative elements, particularly early mobilization and oral intake, and some studies have even found their use to be associated with better quality of recovery or fewer complications. Nevertheless, evidence remains limited by heterogeneity of interventions and variable outcome definitions [57].

Wearable technologies are being increasingly used for continuous monitoring of vital signs, heart rate variability, sleep and activity patterns throughout the perioperative period, extending even into the later recovery period after discharge [58,59,60,61]. Overall, in terms of practical application, the current evidence suggests that wearables and digital platforms are useful for objective measurement of activity, symptom surveillance, and continuous monitoring of vital signs. However, successful and comprehensive implementation into well-integrated ERAS workflows would require defined clinical thresholds and clear responsibility for alert response, filtering of data noise, strategies for alarm burden minimization, and cost and fidelity analyses.

### 5.4. AI and Predictive Analytics

Artificial intelligence (AI) computing, including machine learning and deep learning modalities, is rapidly advancing medical care by offering analytical support in handling complex, high-volume data [62]. In the context of perioperative care, it may play an essential role in bringing together multiple sources of patient-specific data, such as traditional demographic and clinical variables, intraoperative physiological and laboratory results, imaging, multi-omics, and wearable-derived data, and behavioral characteristics, into a single coherent clinical and perioperative profile, which is needed to provide precision perioperative care [63].

AI predictive analytics has been investigated for specific perioperative prediction tasks, but the evidence remains outcome- and context-dependent. For postoperative acute kidney injury, the CMC-AKIX model used preoperative clinical data to predict AKI risk and proposed a web-based decision-support format [64]. Broader reviews describe applications of machine learning for prediction of surgical complications, including current applications, limitations, and implementation implications [65], while other work emphasizes external validation, interpretability, and workflow integration [66]. Furthermore, AI-based predictive models have shown promising performance in perioperative risk stratification. The MySurgeryRisk platform has demonstrated an area under the ROC curve of 0.82–0.94 across several postoperative outcomes [67]. Moreover, an explainable XGBoost model incorporating classical preoperative risk factors and intraoperative physiological and ventilatory variables was reported to predict postoperative pulmonary complications (PPC) with an AUROC of 0.878 in the validation cohort and 0.881 in the prospective cohort, while ARISCAT achieved an AUROC of 0.496–0.533 on the same dataset [68]. In colorectal surgery, a recent systematic review and meta-analysis found promising discriminatory performance of machine learning models for outcomes such as anastomotic leak, mortality, prolonged hospital length of stay, and surgical site infection, but also emphasized that prospective validation, real-time implementation, and evidence of improved clinical outcomes remain limited [69]. Thus, AI-based risk prediction represents a promising tool for precision perioperative care, but it should currently be framed as decision support rather than as a validated mechanism for autonomous dynamic ERAS adaptation.

Another emerging utility of advanced computing is in the area of AI-assisted decision-making, where it is being investigated as a potential support tool for personalized anesthetic and surgical intraoperative management. The use of closed-loop anesthetic dose adjustment through EEG-based depth-of-anesthesia monitoring, or hemodynamic support by integration of hypotension prediction and organ injury risk assessment, has demonstrated technical feasibility, but evidence regarding postoperative outcomes remains inconsistent [70].

In the context of ERAS, AI use may not be limited to risk estimation and decision support. Although chatbots and digital assistants are not precision medicine interventions in the strict sense, they may support patient education, guidance, and engagement, while their impact on adherence, safety, and clinical outcomes remains to be established. Preoperative counseling is an integral principle of the ERAS pathway [12], as it creates the grounds for meaningful patient integration and adherence in the recovery process. Such tools can make complex medical information more accessible while reducing the burden on healthcare staff and optimizing time management [56,71].

It is also important to note that another proposed advantage of AI in perioperative care is its ability to continuously update the patient’s profile through a continuous data stream, which potentially allows for care to transition from static observations to a dynamic and responsive adaptation during recovery. However, current integration is limited by several factors. Most AI models are developed using retrospective, single-center datasets with limited external validation, raising concerns about robustness across diverse patient populations and healthcare systems. Regulatory and ethical considerations further complicate implementation, including issues related to data privacy, algorithm bias, and medico-legal responsibility. Therefore, future integration is dependent on the development of transparent, interpretable models; robust prospective and randomized validation; standards in data architecture; and thoughtful workflow integration [72].

A conceptual summary of the findings is provided in Table 1.

## 6. Conceptual Model of “Precision” ERAS

**Figure 2:**
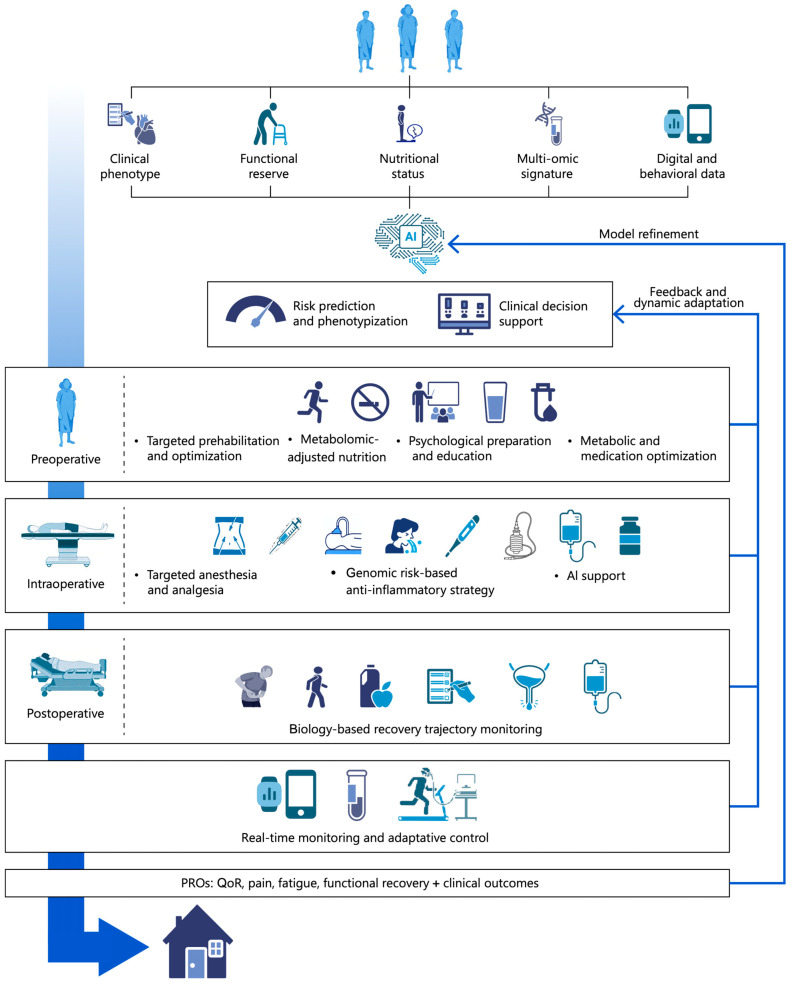
A conceptual model of a precision ERAS framework. The figure illustrates a proposed layered model in which standard ERAS care remains the foundation, while additional patient-specific data streams may support future risk stratification, perioperative adaptation, monitoring, and feedback. The model should be interpreted as a research roadmap rather than an operational clinical pathway. Several components, including multi-omics profiling, advanced predictive analytics, wearable-derived phenotypes, targeted anti-inflammatory strategies, and adaptive feedback systems, remain investigational and require prospective validation, predefined decision thresholds, evidence-based intervention triggers, and assessment of clinical utility before routine implementation. PROs—patient-reported outcomes. Created in https://BioRender.com, accessed 1 May 2026.

An idealized conceptual model of precision ERAS should build upon but not replace the foundational principles of standard ERAS care. As illustrated in Figure 2, a precision ERAS model preserves the core ERAS principle of standardized, evidence-based perioperative care while suggesting how selected elements could be adapted to individual patient biology, physiology, functional reserve, and recovery trajectory. We propose a conceptual framework for such precision ERAS model, which should be primarily viewed as a research roadmap for future scientific exploration, hypothesis generation, and prospective validation rather than as a ready-to-implement clinical pathway.

At its foundation, the model remains grounded in established ERAS interventions, including patient education, preoperative optimization, multimodal analgesia, goal-directed fluid therapy in selected patients, early mobilization, early oral intake, and standardized discharge criteria. This is important because the current evidence still supports ERAS primarily as a structured, protocol-driven intervention. The precision component should therefore be viewed as an additional layer applied selectively rather than as a replacement for ERAS fidelity.

The precision approach is organized into layers of data acquisition, data integration, risk prediction, targeted perioperative adaptation, dynamic postoperative monitoring, and feedback-based refinement. The first proposed layer is multidimensional data integration. In an ideal precision ERAS system, diverse patient-specific inputs such as clinical phenotype, comorbidity burden, frailty, functional reserve, nutritional status, inflammatory markers, pharmacogenomic information, selected biomarkers, wearable-derived activity or physiological data would be synthesized into a coherent perioperative profile, which would guide further planning and precision risk adjusted care.

In the second layer of prediction and stratification, integrated data would allow clinicians to ideally estimate not only the probability of complications, but also the mechanisms through which risk is generated and the interventions most likely to reduce that risk. At present, this goal has only been partially achieved. Existing risk scores and predictive models can identify high-risk patients, but they often provide limited guidance on which specific ERAS element should be intensified, withheld, or modified.

Based on the patient’s predicted biological risk, dynamic personalization of ERAS elements would allow the adaptation of timing, intensity, and combination of interventions, such as prehabilitation, nutritional optimization, anesthetic management, analgesia, fluid therapy, postoperative monitoring, and mobilization.

Within the fourth layer of dynamic monitoring and adaptation, continuous perioperative data streams would potentially allow the update of the patient’s risk profile in real time and detect deviations from the expected recovery trajectory, thus enabling earlier escalation or safe de-escalation of care.

The final layer is feedback learning, in which patient outcomes, ERAS compliance data, complications, patient-reported recovery, readmissions, and resource use would be fed back into the system to refine future prediction and decision-making. Although audit and feedback are already central to successful ERAS implementation, a true learning precision ERAS system would require interoperable electronic health records, standardized data definitions, validated predictive models, governance structures, privacy safeguards, and continuous monitoring for bias, safety, cost, and equity.

Importantly, the greatest potential benefit of precision ERAS is unlikely to occur in low-risk surgical populations, where standard ERAS pathways already achieve excellent outcomes [12]. Instead, current evidence suggests that biologically vulnerable patients continue to experience high complication rates despite ERAS implementation [73]. These populations include frail older adults [74], patients with sarcopenia or malnutrition [75], individuals with chronic organ dysfunction [74,76], and those undergoing major abdominal surgery [73]. Persistent postoperative morbidity in these patient groups suggests that standardized pathways alone may not fully account for patient-specific variability in surgical stress responses and recovery trajectories. Consequently, these populations represent logical initial targets for future precision ERAS research.

To more clearly define the clinical actionability of the proposed framework, a hypothetical frail older patient undergoing colorectal surgery can be used to illustrate how precision ERAS may add value beyond the clinical individualization already embedded in contemporary ERAS pathways. In such a patient, standard ERAS care would already include individualized planning based on age, comorbidity burden, frailty, anemia, nutritional risk, renal function, cardiopulmonary reserve, medication profile, and functional status. Therefore, the added benefit of precision ERAS would not be the recognition that the patient is frail, nor the routine adaptation of care according to clinical judgment. Rather, the precision component would arise from integrating additional patient-specific biological, pharmacological, digital, and predictive data that refine risk estimation, identify mechanisms of vulnerability, and trigger predefined perioperative actions.

Genomic data could theoretically be used in identifying biological vulnerability that is not captured by conventional frailty or comorbidity assessment. For example, genetic susceptibility to postoperative delirium, exaggerated inflammatory responses, thrombosis, or organ injury could help identify frail patients whose risk is driven not only by reduced functional reserve but also by specific biological pathways. In such a patient, genomic information would not currently mandate a routine change in ERAS care, but could support future phenotype-based enrichment of research cohorts and closer surveillance for specific complications.

Pharmacogenomics is currently a more clinically plausible precision tool because it can be linked directly to perioperative medication selection. In a frail colorectal patient expected to require opioid-sparing analgesia and antiemetic prophylaxis, CYP2D6 phenotype could inform avoidance of codeine or tramadol in poor, intermediate, or ultrarapid metabolizers, and could support selection of alternative analgesic or antiemetic strategies. This would add value beyond standard ERAS analgesia by moving from empirical dose adjustment to genotype-informed prescribing.

Proteomic and metabolomic data could add value by capturing the patient’s dynamic biological state before or after surgery. Unlike static clinical risk scores, these approaches may reveal early renal tubular stress, endothelial dysfunction, systemic inflammation, immune activation, mitochondrial dysfunction, or altered metabolic recovery patterns. In a frail colorectal patient with chronic kidney disease or high predicted risk of acute kidney injury, a validated renal stress biomarker could trigger a kidney-protection bundle, including avoidance of nephrotoxins, closer hemodynamic optimization, individualized fluid and vasopressor management, glycemic control, and intensified postoperative renal monitoring. Metabolomic profiling may also help identify patients with delayed gastrointestinal recovery or altered metabolic response to surgery, although these domains remain largely investigational and cannot yet be used to guide routine clinical decisions. Thus, the strongest near-term role of proteomic or biomarker-based precision care is not broad multi-omic profiling, but selected biomarker-actionable strategies in which a test result is linked to an evidence-based intervention.

Artificial intelligence and predictive analytics could integrate clinical, laboratory, intraoperative physiological, and postoperative data into individualized risk estimates that are more dynamic than conventional preoperative scores. For a frail colorectal patient, an AI-supported model could update risk for acute kidney injury, pulmonary complications, delirium, prolonged ileus, ICU requirement, or delayed discharge as new intraoperative and postoperative data become available. These outputs could support targeted escalation, such as intensified respiratory physiotherapy, closer hemodynamic monitoring, early geriatric review, or delayed discharge despite apparent fulfillment of some standard ERAS milestones. However, AI prediction should not be presented as autonomous decision-making. Its value depends on external validation, calibration in the local population, explainability, clear thresholds, and predefined clinical response pathways.

Digital health and wearable technologies could be beneficial by transforming recovery assessment from episodic clinical observation into continuous or near-continuous monitoring. In a frail colorectal patient, baseline step count, sleep, heart rate variability, postoperative mobilization, symptom reporting, oral intake, and patient-reported recovery could be used to define an individualized recovery trajectory. A deviation from this trajectory, such as failure to mobilize, persistent tachycardia, reduced activity after discharge, or worsening patient-reported fatigue and pain, could trigger earlier clinical review. This adds value beyond standard ERAS because it measures recovery objectively and longitudinally rather than relying only on ward rounds or fixed postoperative milestones. However, digital monitoring remains supportive rather than definitive; its clinical benefit depends on validated alert thresholds, patient adherence, data quality, and clear assignment of responsibility for responding to alerts.

This scenario illustrates that the actionability of precision ERAS depends on the presence of a clear data-action link. Clinical frailty, anemia, nutrition, renal function, cardiopulmonary reserve, and functional status remain essential data for current individualized ERAS care. However, pharmacogenomics, selected biomarkers, AI prediction, and digital monitoring may add value when they provide actionable information beyond standard assessment. In contrast, genomics, broad proteomics, metabolomics, and advanced wearable-derived phenotypes should currently be regarded mainly as research tools unless they are linked to validated thresholds and predefined interventions. This distinction is important to avoid overstating precision ERAS as a ready-to-implement pathway.

Therefore, the current transformation from standard ERAS to precision ERAS should be described as incomplete and stepwise. The field has moved beyond uniform care toward risk-adapted and patient-centered ERAS, particularly through frailty assessment, anemia correction, nutritional optimization, procedure-specific analgesia, selective goal-directed therapy, and digital adherence support. However, it has not yet reached a mature precision medicine model in which molecular, digital, and predictive data routinely determine specific perioperative interventions.

## 7. Challenges of the Precision ERAS Model

Although the concept of precision ERAS appears attractive, several challenges must be addressed before its widespread adoption in either research or clinical practice. The first challenge reflects the concern that excessive personalization may compromise the simplicity, reproducibility, and scalability that underpin the success of traditional ERAS pathways. Standardization remains one of the principal mechanisms through which ERAS improves outcomes, reduces unwarranted variation in perioperative care, and facilitates reliable implementation across institutions [77,78]. Excessive personalization risks introducing ambiguity, inconsistent decision-making, and reduced pathway fidelity. This challenge may be addressed by maintaining predefined guardrails around core ERAS elements. In practical terms, foundational interventions such as patient education, multimodal analgesia, early mobilization, and early oral nutrition should remain universal, while adaptable components, including prehabilitation, nutritional optimization, hemodynamic monitoring, postoperative surveillance, and analgesic requirements, could be personalized according to patient-specific biological risk profiles.

Implementation represents another major challenge. Experience from precision medicine initiatives in oncology, cardiovascular medicine, and genomics-guided care demonstrates that translating predictive tools into routine clinical practice is considerably more difficult than demonstrating their analytical validity [79,80,81]. Common barriers include insufficient data infrastructure, limited interoperability between electronic health record systems, inadequate clinician training, uncertain reimbursement mechanisms, and the absence of clear evidence that improved prediction consistently translates into improved clinical outcomes [79,80]. Resource requirements may be particularly burdensome for smaller hospitals and healthcare systems with constrained infrastructure [81]. Consequently, successful implementation of precision ERAS would require robust digital systems, validated clinical algorithms, multidisciplinary education, governance frameworks, and continuous quality assurance mechanisms.

Poorly implemented precision systems may inadvertently increase workload, generate alert fatigue, fragment care pathways, and divert attention from interventions with established benefit [82]. Therefore, implementation should remain multidisciplinary and clearly governed. Predictive outputs should not function as autonomous decision-making tools but rather as clinical support mechanisms interpreted within the context of multidisciplinary expertise. The anesthesiologist may lead physiological optimization, analgesic planning, hemodynamic management, and organ protection strategies, while the surgeon integrates risk information into operative planning and procedural timing. ERAS coordinators and specialist nurses may oversee pathway adherence and recovery milestones, whereas dietitians, physiotherapists, pharmacists, geriatricians, intensivists, and pain specialists would become involved when predefined triggers are activated. Clear ownership of both predictions and resulting interventions is essential to avoid fragmented care and unclear accountability.

The integration of precision medicine tools into perioperative care also raises concerns regarding equity and accessibility. Evidence from genomics and precision medicine programs suggests that underrepresentation of certain populations within clinical and molecular datasets may reduce predictive performance among disadvantaged groups [83,84]. Furthermore, patients treated in resource-limited institutions may lack access to advanced diagnostics, digital monitoring technologies, or specialized multidisciplinary services required for precision-based interventions [79,83]. Without careful implementation, precision ERAS could unintentionally widen existing socioeconomic disparities in perioperative outcomes. Future research should therefore evaluate scalability, affordability, accessibility, and patient-reported outcomes alongside traditional clinical endpoints. Equity should be considered a core implementation outcome rather than a secondary consideration.

Artificial intelligence is likely one of the key enabling technologies of precision ERAS; however, its limitations remain substantial. Although machine learning algorithms have demonstrated notable predictive performance in perioperative risk assessment, predictive accuracy alone does not guarantee clinical utility [85,86]. AI systems remain vulnerable to algorithmic bias, limited transparency, poor explainability, and reduced generalizability when applied to populations different from those used during model development [83,84,85]. Studies from broader healthcare settings have demonstrated that AI models may perform differently across demographic and socioeconomic groups, potentially perpetuating or amplifying existing inequalities embedded within training datasets [83,84]. Furthermore, performance may deteriorate over time because of changing clinical practices, patient populations, and healthcare environments, a phenomenon known as model drift [87].

Another important concern is automation bias, whereby clinicians place excessive trust in algorithmic recommendations despite conflicting clinical evidence or judgments [88]. Besides alert fatigue—a well-recognized limitation of clinical decision support systems—excessive generation of risk scores, notifications, and recommendations may paradoxically reduce clinician engagement with genuinely important alerts [82,89]. To minimize these risks, future AI-enabled precision ERAS systems should prioritize explainability, transparency, and actionability. Prospective validation studies, external validation across diverse healthcare settings, continuous monitoring for model drift, periodic recalibration, and multidisciplinary oversight should be considered mandatory before widespread clinical implementation [85,86,87].

Finally, the risks of data overload and false precision must be acknowledged. The availability of large volumes of physiological, molecular, and behavioral data does not necessarily translate into clinically meaningful improvements in decision-making [80,81]. Rather than indiscriminately collecting increasingly complex datasets, precision ERAS should focus on identifying the minimum validated dataset necessary to improve specific perioperative decisions. Similarly, predictive algorithms should undergo rigorous evaluation of discrimination, calibration, clinical utility, and implementation effectiveness before integration into routine care [85,86,87]. Robust governance structures, transparent regulatory oversight, and comprehensive privacy safeguards will be essential to protect sensitive patient information and ensure the ethical deployment of advanced precision technologies [72].

## 8. Research Priorities for Precision ERAS

To create the opportunity to implement a realistic precision ERAS model, we will need high-quality evidence from prospective, multicenter and pragmatic trials evaluating whether such a precision model informs interventions and translates into meaningful patient-centered outcomes, including functional recovery measures, quality of recovery, health-related quality of life, return to daily activities, and postoperative pain burden. Research should also aim to define and validate perioperative phenotypes by integrating clinical, biological, and digital data to better understand variability in surgical stress response and recovery.

A major research priority is to move from broad conceptual risk stratification toward clinically actionable decision frameworks. Future studies should define the minimum required variables needed for a precision ERAS assessment, including which clinical, physiological, laboratory, functional, digital, or biological data elements are essential, feasible, and reproducible across different healthcare settings. Similarly, validated decision thresholds are needed to determine when a predicted risk profile should trigger a specific perioperative response. These thresholds should be prospectively tested and linked to predefined actions, such as intensified prehabilitation, nutritional optimization, enhanced hemodynamic monitoring, altered analgesic strategies, postoperative surveillance, or escalation to multidisciplinary review.

Another unresolved issue is the management of conflicting predictions. Different tools may identify divergent risks in the same patient; for example, frailty assessment, inflammatory biomarkers, wearable-derived functional data, and machine learning models may provide discordant estimates of postoperative vulnerability. Future research should therefore evaluate how such conflicts should be interpreted, prioritized, and translated into clinical decisions. This may require predefined decision hierarchies, multidisciplinary adjudication, or conservative safety-based escalation when uncertainty is high. Importantly, each output of a precision ERAS model should be linked to a clearly specified clinical action, responsible team member, and measurable outcome instead of remaining a descriptive risk estimate.

In addition, the development and external validation of machine learning predictive models, together with careful planning of data architecture, interoperability, data governance, and data safety, will be of critical importance. Integrating implementation science methods and health–economic analyses is necessary to ensure that the model remains feasible, scalable, and equitable, particularly in resource-limited settings.

AI may also support more efficient and accelerated research. Big-data and machine-learning approaches may support faster pattern identification in healthcare datasets [90]. Real-time risk modeling could, in principle, support more adaptive research, where models are refined dynamically, although prospective validation and governance remain essential [63]. However, faster models do not necessarily equate faster implementation, as prospective validation, regulatory approval, financial analyses, and workflow integration remain time-consuming though essential steps. This mandates a careful and stepwise approach to the precision ERAS model.

## 9. Conclusions

ERAS has provided an effective evidence-based framework for improving perioperative outcomes, yet persistent variability in complications, functional recovery, and patient-centered outcomes suggests that protocol adherence alone cannot fully address patient heterogeneity. Precision medicine may therefore complement, rather than replace, ERAS by enabling selected pathway elements to be intensified, modified, or de-escalated according to biological risk, response, and recovery trajectory.

Several individualized components are already embedded in contemporary ERAS practice, including risk-adapted optimization, anemia and nutrition management, goal-directed hemodynamic therapy, patient- and procedure-specific analgesia, and functional recovery goals. More advanced tools, such as pharmacogenomics, multi-omics, wearable monitoring, and artificial intelligence, remain promising but insufficiently validated for routine use. Their implementation is currently limited by uncertain clinical utility, interoperability barriers, costs, workflow disruption, and potential inequities between healthcare systems. Before precision ERAS can be recommended as a fully integrated clinical pathway, prospective multicenter studies, pragmatic trials, external validation of predictive models, health–economic analyses, and implementation research are required. At present, precision ERAS is unlikely to be broadly implementable as a complete model, although selected components may be introduced stepwise within established ERAS programs.

As a narrative review, this paper is limited by the absence of a systematic strategy and formal study selection process and therefore cannot claim comprehensive or reproducible evidence synthesis. Nevertheless, it provides a conceptual and pragmatic framework for future research.

## Figures and Tables

**Table 1:** Emerging precision medicine tools relevant to precision ERAS.

Domain	Level of Evidence/ Clinical Readiness	Potential Future Application Within ERAS	Potential Impact on Patient Care	Key Limitations/ Challenges
Genomics [33,34,35,36]	Exploratory/Research-only readiness	Identification of genetic risk for complications (e.g., AKI, thromboembolism, and delirium)	Refine biological risk phenotyping	Cost; limited availability; need for validation
Pharmacogenomics [38,39,40,47]	Moderate/Selectively actionable	Genotype-guided selection or dosing of perioperative drugs	Reduce adverse drug reactions; optimized analgesic choice	Turnaround time; implementation planning
Proteomics [48,51,52,54]	Emerging/Selectively biomarker-actionable	Early detection of organ dysfunction	Support earlier surveillance or biomarker-guided intervention	Standardization and clinical integration
Metabolomics [49,50,55]	Exploratory/No validated thresholds	Prediction of poor recovery and complications	Support identification of metabolic recovery patterns	Complexity of data interpretation
Digital health [58,59,60,61]	Emerging/Uncertain outcome benefit	Monitoring mobility, sleep, and recovery after surgery	Improve patient engagement and objective recovery assessment	Data reliability; patient adherence
Remote monitoring [59,60,61]	Emerging/Requires validated alerts	Early detection of deterioration in surgical wards	Support earlier recognition of deterioration	Alarm fatigue; infrastructure needs
Clinical decision support [63,66]	Emerging/Requires workflow integration	Real-time recommendations within ERAS	Improve consistency of risk-adapted care	Workflow integration challenges
Predictive analytics [30,64,65,67,69]	Emerging/Requires prospective validation	Dynamic adaptation of ERAS pathways	Support risk stratification and selective escalation of care	Data interoperability issues, model drift
Precision individualized anesthesia [67,68,70]	Emerging/Outcome evidence inconsistent	Personalized fluid, vasopressor, and anesthetic management	Reduce physiological instability in selected cases	Cost; clinician training

AKI—acute kidney injury.

## Data Availability

No new data were created or analyzed in this study. Data sharing is not applicable to this article.
